# The Walkshop Approach to Science and Technology Ethics

**DOI:** 10.1007/s11948-014-9526-z

**Published:** 2014-02-06

**Authors:** Fern Wickson, Roger Strand, Kamilla Lein Kjølberg

**Affiliations:** 1 GenØk Centre for Biosafety, Forskningsparken, PB 6418, 9294 Tromsø, Norway; 2 Centre for the Study of the Sciences and the Humanities, University of Bergen, PB 7805, 5020 Bergen, Norway

**Keywords:** Ethics, Science, Technology, Responsible innovation, Environment, Walkshop

## Abstract

In research and teaching on ethical aspects of emerging sciences and technologies, the structure of working environments, spaces and relationships play a significant role. Many of the routines and standard practices of academic life, however, do little to actively explore and experiment with these elements. They do even less to address the importance of contextual and embodied dimensions of thinking. To engage these dimensions, we have benefitted significantly from practices that take us out of seminar rooms, offices and laboratories as well as beyond traditional ways of working and interacting. We have called one such practice the ‘walkshop’. Through walkshops, we have spent several days walking together with our colleagues and students in open outdoor spaces, keeping a sustained intellectual discussion on ethical aspects of science, technology and innovation while moving through these landscapes. For us, this has generated useful opportunities to escape established hierarchies, roles and patterns of thought and to rethink conceptual and philosophical issues from new perspectives, under new attitudes and with renewed energy. In this paper we wish to highlight the potential benefits of the walkshop approach by sharing some of our experiences and describing how we have prepared for and carried out these events. We share this information in the hope that we may encourage others to both experiment with the walkshop approach and exchange information on their own innovative processes for research and teaching in science and engineering ethics.

## Introduction

The social and ethical aspects of modern science, technology and innovation are currently experiencing an increased level of attention within policy circles. National and supranational research policies now regularly call for “socially robust” or “responsible” research and innovation in which the processes of science and technology development are explicitly expected and encouraged to identify and address their social and ethical dimensions.^1^ This is particularly the case in emerging sciences and technologies (such as biotechnology, nanotechnology, information technology, cognitive science and the various fields of interaction between them) where the potential for social controversy is seen to challenge the future development of the field and/or an acceptance of the innovations it may generate. To date, one of the primary means promoted for achieving more socially robust science and/or responsible innovation has been the sponsoring of research and teaching on “ELSI/ELSA”^2^ within large-scale investments in science and technology (Zwart and Nelis 2009), as well as encouraging increased interaction and integration across the social and natural sciences (Stegmaier 2009). This has seen ever more ethicists, philosophers, social scientists and lawyers enrolled in research and teaching activities connected to science and technology programmes.

In a quest for a consideration of social and ethical aspects that does not arrive too late to influence the decisions that shape scientific and technological developments, methodological discussions within ELSA have currently tended towards precautionary and participatory forms of technology assessment (van Est and Brom 2012), models for anticipatory governance (Guston and Sarewitz 2002), practices of socio-technical integration and ‘midstream’ modulation (Fisher et al. 2006; Schuurbiers 2011) and modes of ‘upstream’ analysis and engagement (Wilsdon and Willis 2004). This emphasis on early consideration of social and ethical aspects often necessitates an analysis of knowledge and technology that may not yet exist as anything more substantial than visions, hopes and imaginations. In order not to naïvely rely on what has been called sociotechnical imaginaries (e.g. see Jasanoff and Kim 2009)—and thereby restrict one’s moral imagination to the expected and desired outcomes of scientific and technological development—ELSA teaching and research has benefited from both the inclusion of perspectives from science and technology studies (e.g. see Jasanoff et al. 1995; MacKenzie and Wajcman 1985) and from pursuing ethical reflection and analysis in direct collaboration with scientists, technology developers and innovators.

The practice of performing research and teaching on the ethics of science and engineering in an integrated or collaborative mode does, however, encounter a range of challenges and pathologies that are not explicitly handled or well addressed in currently dominant ELSA methods. These include: (1) insufficient time and attention dedicated to unearthing and understanding the competing worldviews, epistemologies and values that can be at play when scholars from the social sciences and humanities interact with those working in the natural sciences, (2) a failure to address the power dynamics, perceptions and projections that operate across the divide between the so-called hard and soft sciences, (3) an historical undercurrent of distrust between practitioners of science and ethics, (4) an inability to challenge existing academic hierarchies and the way that these can inhibit open conversations and, (5) a lack of recognition of the significant role that space, place and the materiality of nature-culture relations play in shaping reflections on social and ethical aspects of science and engineering.

As researchers and teachers interested in the ethics of science and engineering, these challenges and pathologies have driven us towards methodological curiosity as we have felt a strong need for new approaches to our work, particularly when this is to be performed in direct interaction with scientists and engineers. Specifically, we have felt that the complex relationship between human understanding and agency on one hand, and the technological, cultural and natural context within which this develops on the other, calls for critical reflection on standard academic practices and modes of operation. For example, when giving a course on environmental ethics, to what extent does it matter if teachers and students spend all their time sitting on plastic chairs inside a seminar room with windowless concrete walls? If the matter of concern is, say, Stephen Toulmin’s (2001) plea for a *Return to Reason* and a sensitivity to particulars, context and concrete details, does it matter if the predominant way of discussing issues is one of ever more sophisticated theory with increasingly higher levels of abstraction? Feeling that these things do indeed matter, we began experimenting with styles of work that broke with some of the standard academic routines: notably getting out of the chairs, out of the seminar rooms, out of the university campus and also out of the city. This paper is devoted to one such set of experiments that we have called *walkshops*.

Although the walkshops we have conducted to date have been varied in form, content and purpose (in line with our experimental aims) some overarching features or contours can be identified as characterising the general approach. In its simplest terms, a walkshop can be thought of as a workshop conducted through walking. It retains the characteristic of gathering a group of people together to analyse and reflect upon a shared topic of concern, but in the case of a walkshop, the majority of the discussion takes place outdoors as the group moves through a landscape. The structure of the program however is purposively more fluid and flexible than a standard workshop, with conversation encouraged to flow on, around and beyond the set topic of concern in an organic and dynamic manner. Walkshops specifically emphasise the importance of spending extended time together as a group and the relevance of this time being used to get to know each other on levels that are deeper or more extended than standard academic encounters facilitate. Furthermore, walkshops actively engage with and use landscapes as stimuli for discussion and reflection.

The value of both using the outdoors and walking as a way to stimulate reflective thinking have been appreciated and documented in various fields for some time. There has, for example, been a long association between the practices of walking and philosophy, first recorded in writing as early as Aristotle’s peripatetics. This connection was, however, significantly strengthened by Rousseau and the Romantics, where it was specifically combined with critical consideration of the role of science and technology in society and the form of nature-culture relations (Solnit 2001). Also within the philosophical tradition, Heidegger and phenomenology have taught that “dwelling” in a place is more than merely observing it and that this can be a matter of human development and identity—perhaps even *Dasein* and authenticity for some. In recent years, human geographers have expanded on this by not only giving particular attention to notions of space and place, but also to the act of walking itself and how it relates to embodied experience and anthropological fieldwork (e.g. Lee and Ingold 2006). Using the outdoors for fieldwork has of course long been recognised by natural historians, biologists and ecologists as providing unique access to certain types of knowledge and understanding, while environmental and outdoor education have also demonstrated long experience with using the outdoors to supplement classroom based teaching, explore environmental values, and facilitate the development of caring ecological citizens (Sandell and Öhman 2010).

Within our own national context in Norway, being outdoors or living the ‘friluftsliv’ (open air life) is a deeply rooted cultural value that has played an important role in the shaping of Norwegian national identity in the 19th and 20th centuries. The Norwegian author Henrik Ibsen coined the term “friluftsliv” and it remains very much in general use as a profoundly positive identity marker of Scandinavian culture (Gelter 2000). This general cultural value has also arguably inspired the development of a specifically Norwegian body of ecophilosophy (Reed and Rothenberg 1993). The idea of “friluftsliv” emphasises the value of enjoying simple outdoor activities such as walking, fishing, picking wild berries etc. and spending time in close contact with nature and directly experiencing non-urban landscapes. The idea being that to experience and enjoy nature on its own terms creates “a diverse range of challenges to the total person, and […] an opportunity for emotional, physical, and intellectual engagement” (Faarlund 1993, p. 163). In addition to ‘friluftsliv’ being a deeply rooted cultural value, Norway is also committed to the value of having ethics included in the training of scientists across all disciplines. Training in the philosophy of science and research ethics is a mandatory component in Norwegian PhD programmes according to the national guidelines of the Norwegian Association of Higher Education Institutions (Universitets- of Høgkolerådet 2013) and competence in the philosophy of science and ethics is also a required learning objective of the PhD according to the Norwegian National Qualification Framework for Higher Education (NOKUT 2014).

However, even if Norway is seen to possess cultural values and a landscape of vast unpopulated moors and mountain areas that may be considered particularly conducive to adopting the walkshop approach to ethics of science and engineering, it is important to note that the contrast we wish to make between the university campus and the mountain trails we have used for our walkshops is not the romantic one of pure culture and pure nature, or between the authentic and inauthentic. The signs of human presence are there to see in the moors and mountains for all who know how to spot and measure them—in the landscapes, in the biodiversity and in the chemical composition. Likewise, the university campus is full of nature in terms of matter, energy, bodies and life forms. What the move from the indoors to the outdoors or from the campus to the mountains offers for us is rather an *altered* nature/culture dynamic, *different* materialities, and a change in degree, going from a relatively static, controlled, secure environment to a more varied, dynamic and challenging terrain. A walkshop is therefore not only a kind of workshop that takes place primarily in the outdoors or the open air but which in doing so thereby draws on different materialities and nature/culture dynamics than those typically experienced in traditional academic settings. In our case, we have used this approach to specifically facilitate reflective mutual learning across different perspectives and around challenging social and ethical issues related to the development of science, technology and innovation.

Aware of traditions connecting the outdoors and education, walking and thinking, nature and culture, we developed the walkshop as something more than just a field trip to learn about nature or place, but also as something more than just a team-building exercise or free-floating philosophical contemplation. Adopting a kind of hybrid identity, our walkshop approach has allowed us to productively change social dynamics, reimagine nature-culture relations, build trust and understanding, and open creative spaces for thinking. More concretely, the value that we have experienced using this approach includes: the ability to use the materiality of a landscape as a tool for facilitating reflection, the capacity to productively alter social dynamics through enabling embodied encounters and challenging existing hierarchies, and the power to alter established patterns of thought through the combination of unmediated outdoor experiences with different social dynamics.

In what follows we will present our experiences with the walkshop approach and how it can be organised and carried out in practice. To do this, we will begin by sharing illustrative anecdotes from three of the different walkshops we have conducted. These stories from practice are told as a way of demonstrating the value of this approach as we have experienced it and showing how it can be a useful new method for exploring science and engineering ethics that overcomes some of the challenges and limitations of existing alternatives, as described above. Following our sharing of these walkshop stories as a way of illustrating the value and potential of the approach, we will impart some of the concrete lessons we have learned for what needs to be considered in the preparation and conduct of a walkshop. In doing so, we aim to provide information in a way that does not prohibit further exploration and experimentation with the general approach by others who may have diverging interests. It is also our sincere hope that our sharing of experiences may give others the impetus to tell their stories of methodological innovation in research and teaching around science and engineering ethics for our mutual benefit. This is important because as science and technology continue to advance in novel directions, we need to ensure that the way we approach research and teaching on their ethical aspects also evolves to remain relevant to new generations of practitioners.

## Stories from Practice: Illustrating the Potential Value of the Walkshop Approach

### “Ecological Ethics, Risk and Governance”: A Walkshop Around Altevatnet Lake

In the autumn of 2011, we held a four-day walkshop for 10 participants in the mountains surrounding Altevatnet lake in the North of Norway. The theme was “Ecological Ethics, Risk and Governance”. This walkshop represented the kick-off meeting of a project funded by the Norwegian Research Council to integrate environmental ethics and ecotoxicology for responsible ecological governance of emerging technologies. The primary motivation behind holding a walkshop rather than a standard workshop in this case was to try and create a strong bond of understanding amongst the interdisciplinary group of researchers funded to conduct collaborative research. That is, to try and understand each other’s different perspectives before we attempted to develop a shared agenda, approach and activities. The value of a walkshop over a workshop in this case was multi-dimensional.

In the first instance, we knew from previous experience that interdisciplinary research requires more time (ideally face to face) than disciplinary research (Carew and Wickson 2010). This is primarily because people in interdisciplinary constellations do not necessarily share a common language or worldview. Therefore to be able to work together, time must be spent developing an understanding of where the other is coming from and where potential deep differences and conflicts (e.g. on the level of ontologies or philosophical outlooks) may lie. In this way, the focus of this walkshop was not so much on consensus building, but rather on more clearly identifying our differences so as to see the potential issues that may inhibit fruitful communication and interaction in the future of the project. Time spent together is also essential for building trust. In this research project, there was an aim to discuss ethical issues faced within a certain field of science (ecotoxicology), how scientific practitioners encountered and handled these ethical challenges, and the implications of this for technology governance. To productively open up such issues within a new group of collaborators, trust was essential and the walkshop approach therefore appealing.

The significance of building trust, particularly in projects in which interdisciplinary groups are seeking to identify, discuss and/or address ethical issues, was highlighted when one of the scientists in this walkshop declared that he had had an ‘aha’ moment in which he realised “you are not ethicists, you are philosophers”. Explaining the significance of the difference, he told us how when he had first arrived at the walkshop, he had come prepared to defend his science from a group of “judgemental ethicists” However, over time he had instead come to experience us as “curious philosophers”. Meaning, his perception of ethics was originally something that typically worked to curtail scientific exploration and innovation. Performing research in a potentially controversial area, he had assumed that the focus of an ‘ethics’ discussion would be on what was wrong with what he wanted to do. With this perception, his attitude was not one open to identifying and exploring potential ethical issues connected with his research, but rather to defend his research from the potential negative implications of such investigations. To be open to having a discussion about the ethical aspects and dilemmas of his work clearly required a relationship of trust. In this case, the trust came when the participant realised that this was not a project or an event designed to judge his research and decide what was right and wrong, but rather to try and identify important challenges and see what an interdisciplinary dwelling with the ethical dilemmas may unearth and unveil. This realisation did not come because he was told this was the intention, rather it emerged through a four-day long process of getting to know the other participants, developing an understanding of their interests and motivations, and experiencing how they engaged him in discussions that did not threaten his work but rather sought to understand its ethical challenges.

A concrete example of how this had unfolded was when the scientist hinted at how the expectations of his funder created an ethical dilemma concerning how to communicate his work. Rather than the following conversation revolving around what was right or wrong for the scientist to do in this case or what might be established as relevant policy recommendations to avoid such situations, the conversation on the mountain trail of the walkshop flowed more freely into reflections over what is being asked of science in today’s world, how this varies with different types of actors and different types of science and the ways in which public and political demands for ‘impact’ and ‘innovation’ are encouraging and facilitating unethical actions. Over time, as the scientist continued to experience this mode of reflective exploration in the walkshop conversations, trust was engendered and his image of other participants as “judgemental ethicists” was remodelled into one of “curious philosophers”.

While it could be argued that trust could also be established through extended time in an ordinary workshop setting, there are important elements of the walkshop approach that we believe actively help to establish it. One of these is the way in which walking naturally encourages a more reflective and explorative style of conversation, but another is that within the walkshop approach, people engage in a range of conversations beyond the set theme, as well as practices beyond traditional academic routines. Indeed, allowing for this is a significant element of the walkshop approach. That is, while the walkshop requires thought and very careful planning so as to facilitate a situation in which conversations can be directed towards the topic of interest, it is equally important that the process is not structured to strangulation and of course, as people walk through the mountains or as they prepare food or sit around a fire at night, they naturally engage in a range of conversations about life in general and their interests beyond academia. Allowing for this, and indeed encouraging it, needs to be given priority alongside the more structured elements of the event because what such an approach encourages is that people (over a relatively short but intense period of time) get to know each other as (multidimensional) people, as opposed to only meeting someone’s academic identity. As one participant put it in an evaluative survey we distributed to participants^3^ “It enables a full immersion in a community, sharing not only the academic work”. This element of the approach actively creates a space in which people who may have fundamentally different philosophies (e.g. on things like the nature of science or the human/nature relation) can come to identify common interests in other areas and build a sense of mutual respect and trust from the base of these other commonalities.

As an illustrative example, we explicitly saw this occur during this walkshop when a social and a natural scientist with very different ideas concerning epistemology and the role of science in political decision-making (differences that may have seen them come to loggerheads and turn away from opportunities for mutual learning in an ordinary workshop format), realised that they shared an interest in wild mushroom collecting. During the days walking, this saw them build a relationship of trust and respect as they hunted for fungal treasures together, shared their knowledge on the different types they encountered, and delighted in each others’ finds. This meant that when discussions in the plenary evening sessions lead into areas of potential fierce disagreement, rather than these blowing up into a debate in which each person attempted to defend an entrenched position, the differences were explored in a way in which there was a genuine attempt to understand the position of the now respected other. Because mutual respect had been built around common areas of interest outside the topic of academic discussion, facilitated by the walkshop setting and by the ability for the conversation and activities of the day to flow over into non-academic areas, the tone of the conversation on the potentially controversial topic was fundamentally changed. As one of our participants put it in the evaluative survey, “hiking together as a group, making sure that everyone is ok, sharing snacks, helping each other over slippery stones and creeks, etc. helps build trust and relations, which in my case makes it easier to approach participants that I was less familiar with before with questions and reflections on the topic. So my experience was that I got to know, and somehow felt I as making friends with people very quickly and this also allowed for a more free discussion on the different topics”.

Since the research project running this walkshop was focused on the ecological governance of emerging technologies and how the notion of environmental harm is conceptualised and approached across science, philosophy and policy, taking this conversation out of a white walled room under fluorescent lights also offered value. That is, we could use the landscape and our movement through it to influence, inform and stimulate the conversation. One of the concrete ways in which we used our movement through the landscape to help shape our thinking in this case came about through our use of a basecamp. From our basecamp cabin on a husky farm by a small river, we made hikes out to other mountain cabins where we would spend a night before returning the next day to our base. This allowed us to travel a particular path and discuss a specific theme and then to travel the same path back the next day discussing the same theme from a different perspective, just as we were walking the same path but seeing the landscape from a different perspective. In our case this was performed, for example, through considering the notion of environmental harm from a philosophical perspective on one day and from a scientific perspective the next, or exploring how to break down dualisms of nature/culture, nature/technology, humanity/nature in one direction, while exploring the work that the dualisms do and their potential value while walking in the other direction.

The ability to actively use the landscape to initiate, inform, inspire and influence the discussions on the selected topic was taken even further in the walkshop we held in Hardangervidda.

### “Quality in Nature and Technology”: A Walkshop Across the Hardangervidda Mountain Plain

In the summer of 2005 we organised a six-day walkshop with 17 participants at and around Hardangervidda, the mountain plain east of Bergen. The event was called “Quality in Nature and Technology” and combined three purposes. First (and foremost) it was an ELSA research walkshop, centred around philosophical perspectives on the concept of *quality.* 4 Secondly, it qualified as a PhD course on philosophy of science and ethics (provided that the PhD students also produced a written essay after the course). Finally, it was also an outreach/public engagement event in that we organized an open meeting on ethical aspects of technology in the village of Tyssedal, which was the final destination of the walkshop. The combination of purposes made this event an ambitious one in terms of budget, planning and the complexity of the logistics. Also in terms of distances, the walkshop was quite ambitious, covering 67 kilometres in changing terrains during the 3 days of hiking with a group of researchers and PhD students from many countries and disciplines, some of which had little mountain experience. In part intended by us, the walkshop had the character of being a physical and mental challenge for many of the participants, where physical and intellectual struggle coincided and in which everybody was ultimately rewarded with arriving at the final destination, at least in the geographical sense.

Out of the three walkshops that we present in this paper, *Quality in Nature and Technology* was the one with the most specific focus on utilising the relationship between locations, movements and topics, and actively mobilising the landscape space to inform, frame and shape discussions. That is, it had a clear and specific intention to directly use the route and the landscapes it passed through as material for reflection. At this walkshop, we wished to take current challenges of the governance of science and technology as the point of departure, then gradually move the discussion towards “eternal” philosophical questions about human-nature relations, and finally return to the present-day problems again. This movement in the theme was therefore explored in parallel with the changing characteristics and qualities of the selected route. This route involved beginning at a busy mountain motel by a main road, followed by a bus ride to the end of a dirt road and a 25 km walk through rapidly changing landscapes to a mountain cabin in the middle of the mountain plain where we stayed for two nights. The next 2 days of our trail then “followed the water”, with a slow descent along rivers to the small, isolated cabin *Tyssevassbu.* This cabin is electrified and bears in this way evidence of huge, though almost invisible, hydropower installations that reside under ground. From Tyssevassbu the last leg of the walk was the steep descent back into the industrial village Tyssedal, where the planned climax of the trip was the use of an old, extremely steep (70 degrees) open-car funicular used by workers and local inhabitants: an ultimate demand for trust in technology. In Tyssedal, we were received by the local industrial museum that hosted our outreach meeting—Tyssedal was among the very first electrified communities of Norway and played a central role in the country’s industrial revolution based on hydropower at the beginning of the 20th century.

This particular route was chosen after careful consideration to stage the movement of attention around changing nature-culture dynamics and materialities, combined with the sense of challenge and overcoming. The degree to which this intention was noted by the participants is difficult to assess, however, the irony of offering participants a ride in an old-looking open cable car after spending so much time on critical perspectives on technology was obvious to all. The content of discussions over the days of the walkshop ranged across topics such as: what is the value of nature and how this is understood and represented not only in environmental policies but also in the practices and trajectories of emerging technologies such as biotechnology, as well as how quality in nature can be understood in the face of technological change and what work concepts such as precaution, ecological integrity and biodiversity conservation perform. Eight years later, when we asked participants from all our walkshops to answer some evaluative questions, several participants from this walkshop expressed that they still thought of it as a personally rewarding and transformative event, e.g. “a very important personal and professional experience that strongly remains with me”, indicating that the combination of the content, context and process reinforced each other to create lasting impressions and learning.

### “Ecological Responsibility and Emerging Technologies”: A Walkshop Down the Aurlandsdalen Valley

In early autumn 2009 we arranged a three-day walkshop where 11 participants walked from Finse (on the mountain plain between Oslo and Bergen) down the steep and beautiful valley of Aurlandsdalen to arrive in the village of Flåm, situated on the edge of a fjord. This walkshop had the theme “Ecological Responsibility and Emerging Technologies”.

This particular walkshop grew out of a feeling of frustration in our academic work and specifically, our work with the concepts of public engagement and responsible innovation. The initial sense of importance and value that we had felt in these concepts as entrance points to core challenges in addressing ethical issues related to science and engineering had started to fade as we approached and reflected upon their implementation in practice. Within our office space, we felt as though perhaps the ideas had become too abstract to have real meaning and practical value. Despite good discussions at our institute and the frequency of seminars and conferences where these concepts were advocated and endorsed, we had an increasing feeling that the real world challenges of implementing these concepts were not being sufficiently considered. At times, we felt that we were working with concepts so disconnected from their implementation that everyone could agree with their importance, but no one could convince us of how to extract their value in practice. One of the key aims of this walkshop was therefore to create an opportunity in which early career researchers interested in these topics could discuss their concerns, critiques and ideas in depth with some of the more senior and most respected researchers that had been working with these issues for decades. We wanted to take these concepts for a walk with this mixed group so as to investigate whether we could work through our concerns and to see whether we would still feel that the concepts were valuable in a setting so different from the university. The walkshop approach was chosen both to allow for extended time for discussions but also to specifically try and avoid the manifestation of the pathology of academic hierarchies in which the senior staff would fall into lecturing the junior participants about the value of the concepts rather than engage in critical reflection and mutual learning together with them.

As indicated earlier, one of the challenges of a walkshop is to facilitate some level of focus on the theme without attempting to control it too much since unconstrained contemplation and conversation facilitates the value of the approach, including its ability to challenge and flatten hierarchies. Within this walkshop we used a range of techniques and approaches to try and achieve this. For example, the topic for the first day was *responsibility.* For this day, we had prepared a number of small laminated cards with different quotes on each saying something interesting about the concept. After the introduction to the day’s topic (see following section on organizational practicalities), these cards were handed out to the participants. The instruction was to either contemplate these quotes in silence or discuss them with each other and to exchange cards throughout the day if/when desired. The cards were therefore used as a tool to help focus the conversation in a subtle and open way. The organisers routinely stopped at different points to allow the group, which naturally spread out over the trail, to reassemble and remingle conversational partners. However, when walking along, people also needed to take their own breaks to do things like adjust backpacks, share snacks, fill water bottles etc. All of these became natural points to exchange quote-cards and thereby consider the concept from a new angle.

Throughout the day, the natural pace of walking, the shared setting of stunning beauty and the laminated theme cards all created opportunities to easily start conversations with new people. This was a significant factor in breaking down academic hierarchies and was important for establishing the feeling that we were one group of fellow wanderers and seekers rather than professionals inhabiting different rungs on an academic ladder. While it could be argued that opportunities and easy ways to begin conversations also occur during traditional academic conferences or workshops, notably in the coffee breaks etc., it is often very confronting and difficult for early career researchers to both approach and get time with more senior and respected academics in such settings. The extended time and occasions available for this brought about by hiking along the same path, and sharing meals and accommodations over several days, is one of the unique values of the walkshop approach. As expressed by two of our participants (one senior, one junior): “I felt that by hiking together I quickly got to know people, and could discuss issues more freely than I typically do in conferences/workshops with people that are new to me” and the “Variable-geometry of walking groups allows greater (less circumstantially or socially-constrained and less psychologically charged) flexibility for self-organisation of discussion partners. Walking culture allows more relaxed and less formal communication”.

When we arrived at our cabin that first evening, we were able to sit around a fireplace in a lavvo and have the group exchange experiences and thoughts from the day. It was very satisfying to see how effectively the cards had worked and to hear how eagerly the quotes were discussed, with the participants full of enthusiasm and insights. Issues that were discussed in this session included the importance of understanding the philosophical history of the concept of responsibility, the significance of working at an institutional as well as an individual level, and the need to consider the socio-economic and political context in which this concept was now being mobilized for science and technology governance. In the sense of overcoming our frustration with the concepts of interest, the walkshop was a success. This was not because we had a particular revelation that lead to a radical new solution. Rather, it was the experience of being able to freely engage in critical reflection and seeing that this did not kill the value of the concepts, but rather generated eager and enthusiastic discussion which made us feel that what we were concerned with in our research did in fact matter. Equally important though was the sensation that in the mountains, where credit points and CVs seemed distant, our work still made sense. Talking about responsible innovation in this environment did not feel irrelevant or meaningless, but rather even more valuable and important. Contemplating our concepts in a different landscape with a shifting mix of conversational partners uninhibited by strong feelings of hierarchical relations re-inspired us that these concepts did indeed have meaning and significance and we experienced that this held as we traversed a different nature-culture materiality.

## Conducting a Walkshop: Lessons Learned

Through sharing these stories from practice, we have sought to provide anecdotal evidence for our claim that the walkshop approach can have value for research and teaching on science and engineering ethics by altering social dynamics, helping to build understanding, trust and respect, facilitating new ways of interacting, changing the contextual space for conversations and connecting to some of the more physical and embodied dimensions of thinking. However, it takes a lot of planning and careful forethought to run a successful walkshop. Preparation is important both for ensuring the safety of participants and in order to extract the full value and get the desired output. Through our work developing and experimenting with the approach, we have learned a range of the lessons for the practical organisation of such events that we wish to share in the hope that these might be useful for others who may wish to try the walkshop approach in their own work and context. In what follows we will therefore highlight elements that are crucial to consider and offer some of our own approaches as illustrations for what can be done to ensure a successful walkshop. A summary of the relevant points is provided in Table 1.

**Table 1:** Lessons learned from practice for running a successful walkshop

	Before	During	After
Coordinating team	Have more than one person involved in the organisation and planning of the event.	Ensure a clear division of labour Have one organizer at the front and one at the back of the walking group Have organisers debrief at the end of each day	Follow through on any promises made concerning outcomes before or during the event Conduct a thorough debrief on the experience and seek to evaluate its value and success in meeting set aims
Participants	Take into account the required physical capabilities of participants Think about the relevant balance in the group (e.g. gender, age) as appropriate for the theme and group dynamics Invite participants early and ensure they are fully informed about what the process involves	Create a process to get direct feedback from participants on how they are feeling (emotionally and physically) throughout the process—and adapt accordingly Be prepared for minor breakdowns and have treatments (e.g. chocolate, whiskey, rest) available as remedies	Give participants opportunities to debrief on how they experienced the event and to share their evaluations
Location	Perform a trial walk of the track Consider how the landscape may relate to and facilitate the content Consider how the trail relates to the required capabilities of participants Understand any potential seasonal challenges Ensure appropriate accommodations and access to the trail	Find places for plenary gatherings (e.g. in the morning and evening) that serve your purpose and will have minimal interference from others outside the group	Show care and respect for the landscape when you leave it (e.g. by not littering, destroying plants etc.)
Intellectual content	Choose a theme/title Choose a focus for each day Consider relevant literature and distribute prior to the event Identify activities or techniques that will help to facilitate your focus	It is important that each day have a clear agenda/theme (e.g. introduced early in the day through concise presentations, exercises or questions) The end of each day should involve an activity with the group in plenary Consider if and how notes will be taken (e.g. on flipcharts in plenary sessions, on smart phone recorders)	Consolidate as much of the relevant content of the discussions as possible directly after the event so as not to lose it and if relevant, circulate amongst participants for additional inputs
Practical information	Distribute a ‘what to bring’ list including recommendation of a weight limit for packs	Before setting off each day, briefly prepare participants by going over the planned route on a map	Establish ways in which the participants can keep in touch and be updated on outcomes (e.g. shared emailing lists, dropbox folders)
Social elements	Consider how you will balance intellectual content and social activities throughout the event Plan activities that can work as icebreakers, trust builders, free time passers etc. in addition to those advancing the intellectual content	Have activities for facilitating social interactions available for participants at the end of the day should they want them—e.g. a deck of cards, yahtzee, a travelling library etc	Consider having a social event to open and/or conclude the walkshop Don’t underestimate the benefits of sharing small luxuries at the end of the event (e.g. spending the night in a hotel, having pizza and beer, enjoying a restaurant meal)
Prepare for the unexpected	Ensure that participants take out medical/travel insurance and that they provide medical information sheets Check mobile reception and plan emergency escape routes for each day’s trail Carry a well equipped first aid kit and emergency communication equipment	Be flexible enough to change the programme should altered weather conditions, participant illness/injury or the emergence of new ideas require it Be attentive and sensitive to potential conflicts or emotional instabilities and be willing to do something about it immediately	Give yourself permission to follow new lines of interest that may have unexpectedly emerged through the process

### Coordinating Team

**Fig. 1:**
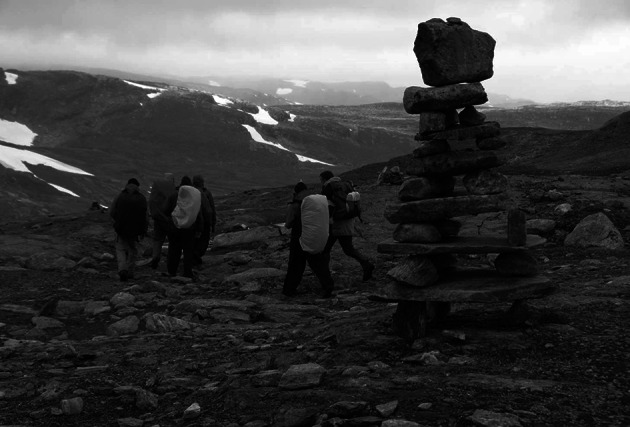
A walkshop in action

We have found it highly valuable to have a multiple-person team to plan and conduct a walkshop. This is important not only because there are a range of organizational elements that need to be in place for the event to work and it is beneficial to share these duties, but also so that organisers can function as a sounding board for each other along the way. During a walkshop it is crucial to be able to be flexible and adapt plans as unexpected situations arise (e.g. as weather changes, as people get ill, as new topics of importance emerge) and having at least one other person to discuss ideas and available alternatives for action with has proven essential. We have structured our events so that each evening the leaders take time to ‘debrief’. This allows the organisers to share opinions about how they think the group is coping, how effective the techniques used for setting the agenda or focusing the conversations are, what might be done to deal with elements that are not working, and to plan how any issues that may have arisen will be tackled in the day to follow. Another relevant lesson for the leaders, organisers or initiators of walkshop events is to ensure that within the organizing group there is a clear division of labour (either for each day or for the event as a whole) and that this division of responsibilities is always clearly communicated to the participants. This division should, for example, include that there is always a leader walking at the front of the group as well as one at the rear and that these roles are exchanged regularly to allow the leaders to also have the possibility to engage in conversations with different participants (i.e. if you are always the person at the front of the group, you will never have an opportunity to discuss the issues of relevance with the slower members of the group, this is also why it is important to stop regularly and allow the group to catch up and shuffle conversational partners) (Fig. 1).

### Participants

The walkshop approach is certainly not something that everyone has the capability or interest to participate in, and therefore the motivation, experience and capacity of potential participants needs to be carefully considered. We have experimented with a range of different formats for the walkshop approach, e.g. hikes over multiple days in difficult mountain terrain, basecamps with overnight hikes across relatively friendly terrain, and basecamps with small day trips into surrounding areas. Both the format and the terrain should be carefully chosen in relation to the participants and their abilities. It is also important to consider the composition of the group, especially considering that in our use of the approach, participants effectively live together for multiple days (spending all day walking together but also sharing kitchens, bathrooms and in our case even sleeping in shared bunkbed rooms in the mountain cabins). While it is not always possible to understand how a group dynamic will function in such a setting in advance, we have found it valuable to actively consider this and to seek a good balance of male/female and younger/older participants. While it is of course possible that a more homogenous group constellation in either age or gender could also function effectively (and indeed may be relevant for certain topics), our experience has been that achieving a balance across these factors can also assist in attaining a balance of personality types and physical capabilities useful for stimulating a harmonious group dynamic and the kind of cooperative and supportive group spirit that is necessary for a successful walkshop. For walkshops taking place over multiple days, we have also found it important not to have too many in the group. We found that 10–15 people was ideal. However, some urban periphery walkshops that have been conducted by a former participant of ours, have successfully engaged around 40 participants. It is important to note though that these were of a shorter duration than those we have run (being in the order of a few hours rather than a few days).

Our experience has been that for the right participants, the notion of a walkshop is incredibly appealing, and it has actually been exceedingly easy to convince outdoor-minded academics to commit multiple days to a walkshop. Despite giving up significant time to participate in such an intensive process, the setting means it rarely feels like work. For example after spending five full days and nights engaged in a walkshop, one senior world-renowned academic stated that the organizers “could have asked for more”, which is rather astounding since this person whose time is in high demand had arguably already given very generously to the process.

One of the important aspects of managing participants during the event is to create a space in which they can give both the organisers and others in the group direct and honest feedback about how they are physically and emotionally feeling. For this we used the idea of a ‘check in’ and ‘check out’. This involved taking a turn around the group at the start and end of each day and inviting participants to share any information they would like, but particularly, to let the group know how they are feeling and what they may want or need out of the day. For example, if a participant is feeling like they would like some time to walk alone and reflect one day, they can communicate this during a check in round so that the rest of the group is aware of their desire. Similarly, if someone has a particular idea or insight they found interesting one day and would like to discuss further, they could mention this during check out so that the next day someone else interested in that topic would know to seek them out along the path.

It is important to note that, especially if walking through challenging terrain and dealing with difficult intellectual content, you can expect at least some participants to have minor meltdowns. Indeed, the check in and out process can serve as a safe space for these emotions to surface. It is important to be aware of the potential for this, not to feel that such things threaten the value of the event, but to be prepared and have potential remedies or relief at hand. Sometimes such remedies may be as simple as some chocolate along the path, some whiskey or red wine in the evening, a shoulder to cry on, an outstretched arm, or a supportive word. The check in and check out process also works to demarcate the period of the day in which formal components of the walkshop take place and therefore when participants are expected to listen to and follow instructions given by the organisers and when they have free time to do entirely as they please.

### Location Selection

The selection of the location to suit your purposes and those of the group is clearly key. Norway is blessed with a vast mountain terrain and a well-connected system of public and private cabins. These cabins are available at three levels of service, e.g. fully catered, food available for purchase, and just the provision of cooking equipment. In our local context then, the choice of trail is partly informed by how serviced we feel the cabins need to be for our particular group of participants and purpose. In other national contexts and landscapes, this question of finding or creating suitable evening accommodations will also be significant for the choice of trails. It is worth noting that the accommodations ideally should also offer a space in which the group can meet in plenary, exposed to minimal interference from others outside the group.

It is also crucial to think of other logistics when selecting the trail, e.g. the ability for people to walk side by side (e.g. a single person width track does not facilitate discussion), the time of year/season and any unique challenges they may represent (e.g. excessive mosquitos), the popularity of the area with other hikers around that time, how accessible the start of the trail is for those coming from abroad etc. As indicated in our example stories from practice, the choice of landscape can also relate to the topic and content. It can be valuable to consider how the landscape and the particularities of where you walk each day may be utilized metaphorically to advance the programme—e.g. trails that are always moving forward into new areas, trails that go back and allow you to see the landscape from another perspective, trails that descend into deep valleys, trails that ascend a peak, trails moving towards more technologically advanced settings etc. We highly recommend that the organisers spend time walking the proposed trail during their preparations to assess its suitability, identify potential problems or highlights, and to specifically think through how the landscape may be used to complement the content.

### Intellectual Content

For the intellectual content it is of course important that the organisers first choose a relevant theme and title for the walkshop to focus on. It is also highly beneficial to give each day a specific theme that can be walked through. In the planning phase, it is crucial to consider what kinds of activities, exercises, presentations, techniques and/or questions may be useful to encourage the group to explore the theme of focus. Our approach has typically been to use some time in the morning after ‘check in’ to set the agenda and theme for the day (either through short presentations, lectures, or a list of relevant questions) and then to gather in plenary again in the evening either to share insights and feedback on what was discussed by different parties during the walk, to conduct particular activities to further reflection (e.g. small group discussions or guided meditation), to try and capture in notes the core lessons of the day, or in the case of educational purposes, to hold further lectures. The issue of how notes from the event are kept is one that needs to be considered in line with the purpose of the event. Since most discussions take place on the trail, it can be difficult to make note of important points raised underway, therefore it can be useful to try and capture and map this (e.g. on flipchart sheets) during an evening plenary. Another option may be to use smart phones to take photos, record conversations, or note down relevant points. Regardless of how notes are taken underway, we recommend that time is also used at the conclusion to consolidate as many of the discussions and insights as possible and that such consolidated notes are circulated amongst the group as a whole so that participants have an opportunity to supplement them with additional information.

### Practical Information

Taking a group of people out into a landscape comparatively isolated from human communities and social services is a large responsibility. It is therefore crucial to prepare participants carefully and well in advance for what a walkshop involves in practice. It is very important that participants receive sufficient information from the outset so that they may know what they are signing up for should they accept the invitation. In this way you reduce the chance of last minute cancellations but also the chance that participants will be shocked by what is involved in practice and allow their struggles to turn into a negative attitude towards the event as a whole.

To aid in this process of giving participants enough information to prepare for the type of event a walkshop is, we have typically provided participants with a list of things they should bring (e.g. rainproof jacket, worn-in hiking shoes, relaxed cabin clothes etc.) as well as things they will not need (e.g. computers, dress clothes, heavy books etc.) and a recommendation of the maximum pack weight they should consider carrying (e.g. 8–10 kg). Prior to the event it is also worthwhile to consider what (if any) reading material may be provided. We have sometimes used novels as required reading (e.g. *The Zen of Motorcycle Maintenance* and *The Road*). These have covered themes of relevance to our walkshops and while they have therefore provided a creative way into the matters of concern and a common reference everyone may refer to, we have also seen them work incredibly successfully as icebreakers for conversation. For example, for the Aurlandsdalen Walkshop, participants were met at an airport and had to then take a 3 h train ride together to the start of the track. Since the participants had not got to know each other at this point and the walkshop had not formally begun, many used the set novel as a way to begin conversations and pass the time on the train ride. Another way in which we have approached reading materials has been to ask each participant to bring something relevant to the theme (which could be a scientific article, a poem, a newspaper article etc.) to contribute to a travelling library that is made available each night should participants wish to read something. While many of our participants have tended to spend the evenings talking together rather than reading from this travelling library, it has often received use in the mornings by early risers looking for a quiet activity to do while waiting for the rest of the group to join them. Since the contributions in the travelling library have been marked with the name of the contributor, this also creates an opening for the piece to be discussed on the trail by those that have read it with those that brought it to the event.

During the walkshop it is also a good idea to spend 5–10 min each morning going over the planned route on a map, highlighting potential stopping points (e.g. for lunch), challenging aspects (e.g. steep sections or river crossings), features of particular natural and/or cultural value etc. In our case we have done this after the agenda setting activities. At the conclusion of the walkshop, it is also valuable to consider how the network of people you have now helped to create may continue to stay in touch. For example sharing an email list, a dropbox folder, new collaborative initiatives etc.

### Prepare for the Unexpected

It is important to be as prepared as possible for any potentially unplanned situations that may occur. In a basic sense this includes carrying first aid kits and having information about participants’ medical history and insurance details. When we have tested the trails during our preparation phase, we have also taken care to identify the areas with and without mobile phone reception and the potential exit routes during each day should something unexpected happen. To perform this level of planning and be able to respond to unexpected events that may arise, it is also advantageous to have more than one person involved. One of the most important elements of being prepared for the unexpected is that the organisers are flexible enough in their thinking and programming to allow for adaptation to occur should weather conditions or illness/injury require it, or if new questions or lines of discussion unexpectedly emerge through the process. Rather than following a strict timetable and programme like a workshop, a walkshop needs to explicitly embrace a more flexible and adaptable attitude. In addition to this flexibility, organisers must also be attentive and sensitive to potential conflicts, emotional instabilities or physical limitations and be willing to take measures to directly address these should they arise.

### Social Elements

As we have already indicated, achieving a balance between focused discussions on the concrete issues and intellectual content of interest and more free-time or social activities where people can meet each other on different terms is crucial. This requires some consideration of the kinds of activities that might be available as icebreakers, trust builders and free time passers (e.g. a deck of cards, a yahtzee set, a travelling chess set or library). At the beginning and/or conclusion of the event we have also found it useful to organize particular social activities (e.g. dinner at a restaurant, a night in a hotel, a pizza and beer party etc.). Such events can be experienced as luxuries, especially after some tough and intense days in the mountains. Indeed having small luxuries available along the way should also not be underestimated as a contributor to the good mood and social interaction (e.g. being able to share a small cognac after a day walking in the rain can be hugely appreciated and stimulate conversation, as can being able to sit around a warm fire).

## Conclusion

For us, the walkshop has become a valuable approach to explicitly engage, contemplate and discuss ethical aspects of science, technology and innovation. Clearly, many forms of academic work place highly specific demands on the physical surroundings—high energy physicists, for example, need laboratory facilities and not an open mountain plain to run their experiments. However, to consider and discuss ethical aspects of science, technology and innovation, we have found significant benefit in changing the traditional settings in which we work. The disciplines of architecture, geography and psychology have all spoken to how our physical surroundings influence human cognitive capacities such as concentration and creativity and yet everyday university life has often given little attention to the physical, spatial and embodied dimensions of academic practices. We have therefore felt the need to directly explore what kind of context is conducive to satisfying our specific academic challenges and needs and here found the walkshop approach to be valuable. As one of our participants expressed it “The context [of the walkshop] reduces academic pathologies. Extended company promotes deeper interpersonal engagement with each other. Landscape provides moderation, punctuation, grounding, poetic inspiration. Broader bandwidth of experience fosters deeper and more vivid recollection. Walking itself adds depth of commitment through exercise bodily involvement enhances shared experience of togetherness”.

The value of the walkshop approach for the specific needs of research and teaching in the ethics of science, technology and innovation has been highlighted by our three stories from practice. In the first instance this included the need to establish good collaborative relationships and social dynamics, above all to build deep trust across disciplinary borders, epistemological positions and moral intuitions. Being together over time, talking, walking and doing practical things can accommodate the need to build trust and communication because one gets to discover more dimensions of the other persons than the narrow role as one’s adversaries, superiors or inferiors in the seminar room. We believe this to be particularly relevant in collaborations that span what Snow referred to as the two cultures (Snow 1959), which is almost always the case in ELSA research and ethics teaching for scientists and engineers. The difference in underlying beliefs and values can make an initial encounter across the two cultures quite a cultural shock in which natural scientists perceive the ELSA researchers as denying and threatening fundamental values and beliefs underlying their work and professional identity. If this relationship is to develop into a productive one in which mutual learning is facilitated, increased attention needs to be given to the process and setting in which the encounter takes place.

Secondly, our own research and teaching has had a particular interest in human/nature relations and the role of science and technology in the ecological challenges we face in late modernity. Here we have specifically utilised the fact that walking through outdoor landscapes offers other stimuli to the senses than indoor sitting. For instance, we believe that we have been able to think differently and perhaps more concretely about values of nature when we can see and move through landscapes with trees, herbs, wild and domestic animals, regulated and unregulated rivers, etc. In this simple way, the surroundings may work as illustration and reminder of certain facts and values as well as an effective stimulus to their reflection and reconsideration. As two of our participants expressed it: “Being in nature…for several days creates a relaxed atmosphere, allowing us to enter into a mode of listening and sharing rather than the daily routine of performing and competing” and “…the method of the walkshop can help us to be more conscious of how we relate not only to nature but to the world and to each other and ourselves.”

Thirdly our experiments with the walkshop approach have shown us how effective the method can be for breaking down established academic hierarchies and allowing participants to meet each other on the more even ground of fellow wanderers in a landscape. In traditional academic settings, established hierarchies manifest pathologies in both the way conversations tend to proceed (with those higher on the ladder typically lecturing on a topic or answering with authority questions posed by those below), and in the ability for those conversations to be opened (with junior scholars typically struggling for time with their elder colleagues and often hesitant or fearful to raise challenging or critical issues). The walkshop approach has proven useful for creating a setting in which these hierarchical pathologies are are challenged from the outset through the use of new kinds of spaces and extended timeframes for which conversations can take place. Additionally, the physical demands of walking on the mountain trails we have used can also help turn these power relations upside down. Our experience has been that changing both the setting for and the style of interactions through using the walkshop approach has substantially counteracted the pathologies of traditional academic hierarchies and lead to a more open, dynamic and creative mode of conversation that offers significant potential and support for processes of mutual learning.

If we do indeed think and work differently during a walkshop as opposed to in an office or a conference room, can one trust the thinking going on in the open air? Where the ethics of science and engineering is interpreted as moral reasoning supposed to produce a final, valid conclusion about the morally correct or sound decision, we have no reason to believe that moral calculation *per se* is more solid while walking outdoors instead of sitting indoors. However, we believe that there are other important elements that could be affected by the thinker thinking differently, by being a different whole person, in a different material setting. These include identifying the *framing* of an issue at hand and exploring alternatives, as well as the importance of cultivating a contemplative, reflective attitude in which our values, choices and assumptions can be subject to critique in a way that is illuminating rather than threatening.

At this point, we could make it simple for ourselves and say that the walkshop works on the context of discovery, while the context of justification and the final validity of arguments may be matters to be assessed in the office or seminar room. Yet philosophically, it may be that our judgements on moral relevance (and by implication ethical validity) do differ when we (phenomenologically speaking) are different. The question then becomes a political one, of how we wish to construct our research and teaching, i.e. as prone to abstraction and meta-theory, giving priority to cleverness in the seminar room, or perhaps more impressioned by vivid interactions with our surroundings and colleagues. Fortunately there is no either-or here and one can do both.

It is important to note that we do not claim that the walkshop is a method that can be used with predictable results for precisely defined purposes. What we do claim, however, is that it can be a valuable approach to teaching and researching the ethical aspects of science, technology and innovation because of the way in which it: facilitates embodied dimensions of thinking, enables colleagues to meet each other as multidimensional individuals, encourages new social dynamics that sponsor understanding, trust and respect, allows new landscapes to enter and shape conversations, and opens creative spaces for dwelling with challenges from new perspectives.

While we have used walkshops for education, exploration and collaboration around the social and ethical aspects of emerging sciences and technologies, some of the values we see in it could clearly also apply to other intellectual challenges and/or fields. For example, the way the approach ruptures counterproductive academic hierarchies, helps to actively build trust, and improve social dynamics may be particularly important for interdisciplinary work on science and engineering ethics, but it would certainly also be valuable in other areas. Additionally, while we have drawn on Norwegian culture and geography and used mountain landscapes, clearly one could also use quite different places and settings (e.g. in cities or on sailing boats) to establish similar procedural conditions. Our aim in this paper has therefore been to present the value we have personally experienced using the walkshop approach and to give some practical guidance for others who may wish to experiment with walkshops in their own work, whether this be within science and engineering ethics or elsewhere.

## Footnotes

1 Examples of this include the Human Genome Project, The US National Nanotechnology Initiative, The European Commission Research Programmes FP5/FP6 (Science-and-Society), FP7 (Science-in-Society) and Horizon 2020 (Responsible Research and Innovation).

2 Ethical, Legal and Social/Societal Issues or Implications (ELSI); Ethical, Legal and Social/Societal Aspects (ELSA). While ELSI was the original acronym used in the US Human Genome Project, ELSA is the preferred concept in European countries due to the flawed linearity seen as implied by the terms issues or implications.

3 In early 2013 when we began to consider writing a paper on the walkshop as a method for teaching and research in science and engineering ethics, we distributed an informal four question survey to all the participants in our previous walkshops to try and gauge to what extent our own reflections and evaluations of the approach and its potential value aligned with their experiences. The questions asked were: (1) How was your experience of participating in a walkshop? (2) What in your opinion are the main advantages and/or disadvantages of the walkshop compared to traditional approaches to academic work? (3) Does anything from your participation in the walkshop remain with you today? If so, do you think the walkshop method had any role in this? and (4) Do you think the method lends itself to particular issues better than to others? If so, what kind of issues? All of the quotes from participants provided in this paper have been taken from the responses to this evaluative survey.

4 The choice of ‘quality’ as a thematic term for this walkshop was inspired by the work of S. O. Funtowicz and J. R. Ravetz, as for example described in their 1990 book, *Uncertainty and Quality in Science for Policy* (Dordrecht: Kluwer).
